# Weight loss in a cardiovascular trial population identifies people at future risk of dementia

**DOI:** 10.1002/dad2.12352

**Published:** 2022-08-30

**Authors:** Pieter van der Veere, Imen Hammami, Georgina Buck, Melanie Greenland, Alison Offer, Michelle Nunn, William Whiteley, Richard Bulbulia, Rory Collins, Jane Armitage, Marion Mafham, Sarah Parish, the Heart Protection Study Collaborative Group

**Affiliations:** ^1^ Clinical Trial Service Unit and Epidemiological Studies Unit Nuffield Department of Population Health University of Oxford Oxford UK; ^2^ MRC Population Health Research Unit Nuffield Department of Population Health University of Oxford Oxford UK; ^3^ Centre for Clinical Brain Sciences University of Edinburgh UK

**Keywords:** cognitive function, dementia, risk prediction, weight loss

## Abstract

**Introduction:**

Populations at increased risk of dementia need to be identified for well‐powered trials of preventive interventions. Weight loss, which often occurs in pre‐clinical dementia, could identify a population at sufficiently high dementia risk.

**Methods:**

In 12,975 survivors in the Heart Protection Study statin trial of people with, or at high risk of, cardiovascular disease, the association of weight change over 5 years during the trial with post‐trial dementia recorded in electronic hospital admission and death records (*n* = 784) was assessed, after adjustment for age, sex, treatment allocation, and deprivation measures.

**Results:**

Among the 60% without substantial weight gain (≤2 kg weight gain), each 1 kg weight loss was associated with a risk ratio for dementia of 1.04 (95% confidence interval, 1.02–1.07). Weight loss ≥4 kg and cognitive function below the mean identified participants aged ≥67 years with a 13% 10‐year dementia risk.

**Discussion:**

The combination of weight loss and high vascular risk identified individuals at high risk of dementia who could be recruited to dementia prevention trials.

## 1 INTRODUCTION

Globally, dementia prevalence has increased from 20 million in 1990 to around 55 million now, and dementia has been cited as the 7^th^ leading cause of death.^1^, ^2^, ^3^ There are no widely available, effective treatments for dementia^4^, ^5^ and so new lifestyle and medical preventative treatments need to be developed and tested in well‐powered randomized trials.^6^, ^7^, ^8^ For example, to be well powered to detect a plausible but worthwhile 20% reduction in dementia risk, a trial of 10,000 participants over 10 years would need to be conducted in a high risk population in which at least 12% of control participants developed dementia (Supplementary Information Table S1, which is available online). So, there is a need to identify populations at higher risk.

Dementia is a clinical syndrome resulting from a number of overlapping pathologies and often has a long pre‐clinical period of 15–20 years or more during which, in addition to cognitive decline, patients may experience a loss of weight, reduction in blood pressure and reduction in activities.^9^, ^10^, ^11^, ^12^, ^13^, ^14^ These reductions could provide an earlier sign of increased dementia risk, and some changes, particularly in weight, can be monitored easily and might be particularly useful as an indication for cognitive testing. Two of the dementia risk profiling measures recommended by the European Task Force for Brain Health Services include a single measure of body mass index (BMI), in combination with other demographic, health and lifestyle factors, but none included weight loss.^15^, ^16^ Although weight loss is associated with a higher risk of dementia over a short timeframe, obesity is associated with greater risks of dementia 15 or more years later.^9^, ^11^, ^17^ Hence, the relationship observed between weight change and dementia is typically non‐linear.^18^, ^19^ Dementia commonly has mixed neurodegenerative and vascular pathology and people with vascular disease are at increased risk of dementia.^20^, ^21^, ^22^ Therefore, high vascular disease risk and weight loss in combination may identify a population at particularly high risk of dementia.

The aim of the present study is to investigate the value of weight change among participants with, or at high risk of, occlusive vascular disease in the Heart Protection Study (HPS) 5‐year statin trial, as a predictor to help identify a population at sufficiently high risk of dementia incidence post‐trial for inclusion in a potential trial of dementia prevention.

## 2 METHODS

### 2.1 Study population

**FIGURE 1:**
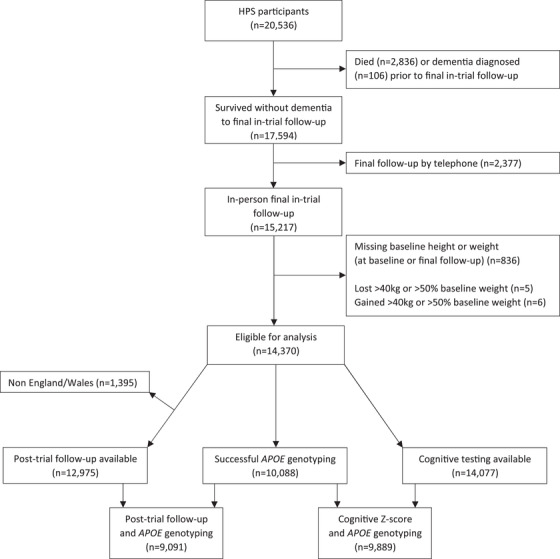
Number of participants included in analyses. *APOE*, apolipoprotein E

The present analyses include participants in the HPS randomized trial of simvastatin versus placebo who survived to a final in‐person follow‐up at the end of the scheduled treatment period (between 2000 and 2001) without a diagnosis of dementia recorded and who had measurements of weight at baseline (1994–1997) and final follow‐up and a measurement of height at baseline (Figure 1).^23^, ^24^ The HPS recruited participants with pre‐existing vascular disease, diabetes, or hypertension from the United Kingdom. Approval was obtained from the ethics committees of the participating institutions and all participants gave written informed consent, including for future medical research.

At regular follow‐up visits until the participant's scheduled final visit, information was sought from them about the occurrence of any serious adverse events. Further information was gathered from additional sources (e.g., hospital records) and more than 99% of participants had complete follow‐up according to the trial procedures. In addition, participants in England and Wales were linked to NHS Digital Hospital Episode Statistics data on hospital admissions, or equivalent the dataset in Wales, and to death registry data with follow‐up until January 31, 2012.

### 2.2 Measurements

Baseline data recorded before randomization included age, sex, smoking, alcohol use, prior disease, current medication use, height, weight, systolic and diastolic blood pressure, and measurements of blood lipid, lipoprotein, and creatinine levels. Weight was measured to the nearest kilogram by the clinic nurse, both pre‐randomization and at the final follow‐up visit a mean of 5.5 (interquartile range 5.1–5.9) years after randomization. Weight change was computed as follow‐up minus baseline weight and percentage weight change as 100 × (follow‐up – baseline weight)/baseline weight. Participants with weight changes more extreme than ±40 kg or ±50% were excluded from the present analyses as outliers (Figure 1). Townsend deprivation index was derived from the participant's postcode at baseline.^25^

Cognitive function was assessed at the final follow‐up visits in 14,077 of the included participants using the 13‐item Modified Telephone Interview for Cognitive Status (TICS‐m)^26^ with an additional verbal fluency test (with both tests generally administered in person by the nurse). The global TICS‐m score together with the verbal fluency score were converted into a global cognitive z‐score using a previously reported procedure.^27^ The cognitive function z‐score was calculated over all HPS participants with cognitive testing and has mean zero and standard deviation (SD) 1 over those participants; its mean and SD in the subset in the present report (with weight measurements) were 0.05 and 0.96, respectively.

Genotyping of two single nucleotide polymorphisms (SNPs) at the *APOE* locus, rs7412 (the ε2 SNP) and rs4420638 (a proxy for rs429358 (the ε4 SNP); r^2^ ∼ 0.7^28^), using a custom I.PLEX panel were available for 10,088 participants in this study.^29^

### 2.3 Ascertainment of dementia

Analyses of dementia were restricted to participants with electronic data linkage (i.e., those from England and Wales). The outcome dementia (and subtypes of dementia) was defined as the first occurrence of an ICD‐10 code diagnosis (in any diagnostic position) in the electronic data following a previously defined specification used in another trial (Supplementary Information Table S2)^30^; Diagnoses in the electronic data prior to final in‐trial follow‐up or the reporting of dementia in the trial (mainly by participants to the nurses at 6‐monthly clinic‐based follow‐up visits, by carers at telephone follow‐up or by general practitioner follow‐up when the former approaches failed^24^) were used to define prior known dementia. Participants with such a diagnosis were excluded from the analysis of post‐trial dementia, leaving 12,975 participants for inclusion in the analysis of dementia incidence (Figure 1).

### 2.4 Statistical analysis

The shape of the relationship between weight change and dementia was investigated since a non‐linear relationship was anticipated on the basis of the previous literature.^17^, ^19^ Therefore, the 14,370 participants eligible for analysis of either dementia incidence or cognitive function were grouped into fifths of weight change, percentage weight change, and baseline and follow‐up weight within each sex. BMI measures were grouped into fifths in the same manner. The bounds for the fifths of each measure are shown in Supplementary Information Table S3, and for weight change were the same in men and women (≤‐4 kg, ‐3 to ‐1 kg, 0—2 kg, 3—5 kg, ≥6 kg). Participants eligible for cognitive analysis were grouped into fifths of residual cognitive function after adjustment for age (as a categorical variable with 20 levels) and sex. Standard adjustment in the analyses of first reported post‐trial dementia was for age (20 levels), sex, randomized trial treatment allocation, height, and Townsend deprivation index. The hazard ratios (HRs) for dementia per 1 kg lower level of weight change across fifths corresponding to weight loss or little change and by groups were estimated by Cox regression. HRs in groups were presented as floating absolute risks relative to the middle weight category (whereby standard errors were assigned approximately independently to each category to avoid restricting comparisons to an arbitrary reference group).^31^ In addition, the associations of baseline and follow‐up weight, percentage change in weight, baseline and follow‐up BMI, change in BMI, baseline and follow‐up systolic blood pressure, change in systolic blood pressure, cognitive function, and *APOE* genotype with dementia were considered in a similar way. Likelihood ratio test χ^2^ statistics for the addition of various measures to standard adjustment were presented to compare the amount of statistical information the measures added. The relationships between *APOE* genotype, cognitive function, and weight loss were also investigated, using linear regression.

The independent predictive value of weight change for dementia was investigated using forward selecting stepwise models over available variables (Supplementary Information Table S4) both with and without the inclusion of cognitive function and *APOE* genotype as available variables (“without” representing the most likely initial clinical situation). To provide some comparison with the published ANU‐ADRI (Australian National University Alzheimer's Disease Risk Index),^32^ BDSI (Brief Dementia Screening Indicator),^33^ and CAIDE (Cardiovascular Risk Factors, Aging and Incidence of Dementia Risk Score)^34^ risk scores, the parts of those scores other than age, sex, and cognitive terms that were available in HPS (Supplementary Information Table S5) were also included as composite available variables for selection. However, only 19/47, 18/33, and 7/11 of the relevant ranges of points in the three scores, respectively, were available in the HPS data.

The absolute observed dementia risk over 10 years within risk categories by age, weight loss, and cognitive function were tabulated. Analyses and plotting used SAS version 9.4 and R version 3.6.2 in RStudio version 1.2.5033.

## 3 RESULTS

Among the 12,975 participants included in the post‐trial dementia analysis, the mean age at the end of the in‐trial period was 69 (SD 8) years, 75% were male, and 97% were self‐classified as of white ethnic origin (Table 1). All participants had prior cardiovascular disease, hypertension, or diabetes by the trial inclusion criteria, and 14.7% had prior cerebrovascular disease at randomization. In trial, 3.0% had an incident stroke, 2.7% an incident transient ischemic attack, and 4.5% an incident cancer recorded. Between baseline and final follow‐up (a mean of 5.5 years later), the mean weight change was +0.9 (SD 6.0) kg, and the mean percentage weight change was a 1.3% increase. About 20% of both men and women lost at least 4 kg, 20% lost 1—3 kg, and 20% gained 6 kg or more (Supplementary Information Figure S1). The characteristics of the 14,077 participants with cognitive assessment were very similar to those with follow‐up for dementia (Table 1).

**TABLE 1:** Characteristics of participants with two weight measurements and post‐trial follow‐up or cognitive assessment at the end of the trial

**Characteristic**	**With post‐trial follow‐up (*n* = 12,975)**	**Cognitively assessed (*n* = 14,077)**
Age at last in‐trial follow‐up in years – *n* (%)		
<70	6411 (49.4%)	7047 (50.1%)
70 to <75	3055 (23.5%)	3309 (23.5%)
≥75	3509 (27.0%)	3721 (26.4%)
Mean (SD)	68.8 (8.4)	68.7 (8.3)
Men – *n* (%)	9728 (75.0%)	10594 (75.3%)
White ethnic origin (self‐reported)^a^ – *n* (%)	12581 (97.0%)	13706 (97.4%)
Baseline measurements – mean (SD)		
Weight (kg)	79.9 (14.0)	79.8 (13.9)
Height (cm)	170.1 (9.0)	170.0 (8.9)
Body mass index (kg/m^2^)	27.6 (4.3)	27.6 (4.3)
Systolic blood pressure^b^ (mmHg)	143.9 (23.1)	143.7 (23.1)
Diastolic blood pressure^b^ (mmHg)	81.4 (11.9)	81.4 (11.9)
Prior diseases at baseline – *n* (%)		
Cerebrovascular disease	1906 (14.7%)	2048 (14.5%)
Peripheral vascular disease	3703 (28.5%)	4251 (30.2%)
Prior myocardial infarction	5291 (40.8%)	5754 (40.9%)
Other coronary heart disease^c^	3007 (23.2%)	3385 (24.0%)
Treated hypertension	5254 (40.5%)	5698 (40.5%)
Diabetes	3863 (29.8%)	3979 (28.3%)
Non‐fatal in‐trial events – *n* (%)		
Stroke	385 (3.0%)	395 (2.8%)
Transient ischemic attack	352 (2.7%)	364 (2.6%)
Myocardial infarction	486 (3.7%)	531 (3.8%)
Heart failure	246 (1.9%)	272 (1.9%)
New onset diabetes	425 (3.3%)	459 (3.3%)
Cancer	584 (4.5%)	613 (4.4%)
Follow‐up minus baseline weight change fifths in kg^d^ – *n* (%)		
≤‐4	2505 (19.3%)	2697 (19.2%)
‐3 to ‐1	2521 (19.4%)	2761 (19.6%)
0 to 2	3126 (24.1%)	3430 (24.4%)
3 to 5	2375 (18.3%)	2567 (18.2%)
≥6	2448 (18.9%)	2622 (18.6%)
Mean (SD)	0.9 (6.0)	0.9 (6.0)

^a^ Other ethnicities were Indian/Pakistani/Bangladeshi (1.5%), West Indian/Guyanese (0.9%), other or mixed (0.5%).

^b^ Baseline systolic and diastolic blood pressure missing in two and three participants with post‐trial follow‐up respectively, and in three and four participants who were cognitively assessed.

^c^ Other coronary heart disease = Stable or unstable angina, or a history of coronary revascularization.

^d^ Boundaries of fifths of weight change were the same in men and women.

### 3.1 Weight change and dementia risk

**FIGURE 2:**
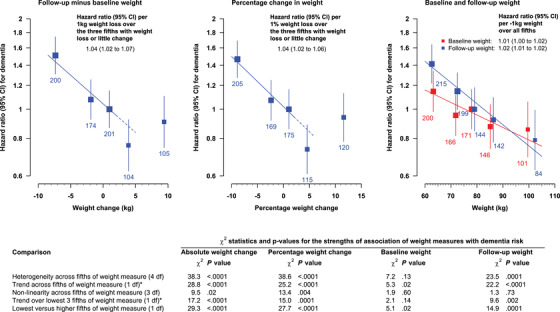
Associations of different weight measures with the incidence of first recorded dementia post‐trial, after standard adjustment. Number of dementia cases shown below each square. The lines displayed in the panels reflect data‐driven summaries of the observed patterns. In the first two panels, the solid line shows the trend over the three fifths with weight loss or little change and the dotted line shows the continuation of this line. *Using the mean weight measure in each group as exposure dose

Dementia was recorded in 784/12975 (6%) participants (including 172 recorded as Alzheimer's, 274 as vascular dementia, and 338 as both or unspecified). There was a non‐linear relationship between weight change and the first recorded incidence of dementia post‐trial (Figure 2). Over the three fifths of the population with weight loss or little change, there was an inverse linear association between weight change and the HR for dementia on a log scale (HR per 1 kg weight loss 1.04, 95% confidence interval [CI] 1.02—1.07), and this relationship extended to the fifth with weight gain of 3—5 kg in whom the lowest risk of dementia was observed. Among those with weight gain ≥6 kg, there was a somewhat higher risk of dementia, similar to that in participants with little weight change. The fifth with the greatest weight loss (≥4 kg) was associated with an HR of 1.51 (95% CI 1.31–1.74) relative to the middle group (weight gain 0–2 kg) and an HR of 1.59 (1.35–1.87) relative to the other four groups combined. The percentage change in weight (with the lowest group corresponding to a weight loss of about 4% or more in men and 5% or more in women) showed a very similar association with dementia to that of the absolute change in weight (Figure 2).

Weight at baseline and weight at follow‐up were inversely associated with dementia risk across all groups, but the associations were weaker (as assessed by the χ^2^ statistics) than those with measures of weight change (Figure 2). Height was only measured at baseline, but based on using this value at baseline and follow‐up, changes in BMI showed similar strengths of association with dementia to those with weight change (Supplementary Information Figure S2). No measures of systolic blood pressure or change in systolic blood pressure were statistically significantly associated with the first recorded incidence of dementia post‐trial (Supplementary Information Figure S3).

Neither the linear associations of weight change with dementia across the three fifths with greatest weight loss, nor the associations of the fifth with greatest weight loss versus other fifths of weight change with dementia showed heterogeneity by age at final follow‐up, period of follow‐up, or *APOE* genotype (all *P*‐value ≥ .05, Supplementary Information Figure S4).

### 3.2 Cognitive function, dementia risk, and weight change

**FIGURE 3:**
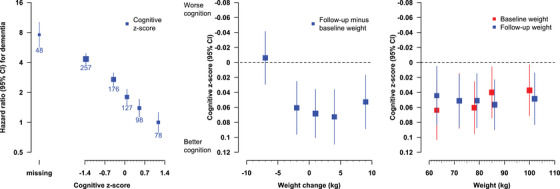
Association of cognitive function with the incidence of first recorded dementia post‐trial and different weight measures, after standard adjustment. In the first panel, the residual of cognitive function z‐score from a model allowing for age (20 groups) and sex is ranked into fifths and the number of dementia cases is shown below each square. Missing = Cognitive function z‐score not available

Cognitive function was strongly inversely associated with dementia risk with a 4.34 (95% CI 3.35–5.61) HR across fifths (Figure 3). The fifth with greatest weight loss was also clearly associated with poorer cognitive function at final follow‐up, but the remainder of the points were consistent with either a trend over the lower four fifths of weight change, as seen for dementia, or a flat relationship between weight change and cognitive function (Figure 3). There was no statistically significant association between baseline or follow‐up weight and cognitive function.

### 3.3 *APOE* genotype, dementia risk, and weight change

Carrying a higher number of *APOE* ε4 alleles was associated with greater risk of dementia (HR 1.98 [95% CI 1.73–2.27]), lower cognitive function z–score (‐0.08 [‐0.12 to ‐0.05]), and greater weight loss between baseline and follow‐up (‐0.40 [‐0.62 to ‐0.18] kg weight change per ε4 allele) (Supplementary Information Figure S5). Carrying a higher number of ε2 alleles was more weakly associated with the opposite effects.

### 3.4 Predictors of dementia risk

In a stepwise selection model for predictors of dementia (with standard adjustment) when cognitive function and *APOE* genotype were not made available, weight change was selected first (Table 2, Supplementary Information Table S4). When cognitive function and *APOE* ε4 genotype were available for selection they were the factors selected first, after which weight change was selected next. Other factors selected (at *P*‐value < .005) were transient ischemic attack in trial and diabetes, insulin use and low‐density lipoprotein cholesterol at baseline. The published risk score composite components were not selected.

**TABLE 2:** Risk factors for the incidence of first recorded dementia post‐trial from stepwise model, considering three scenarios of availability of cognitive test data and *APOE* genotype

		**Without *APOE* and cognitive Z‐score**			**Without *APOE*, with cognitive Z‐score**			**With *APOE* and cognitive Z‐score**		
**Factor**	**DF**	**HR (95% CI)**	**Step**	**χ** ^2^ **change from previous step**	**HR (95% CI)**	**Step**	**χ** ^2^ **change from previous step**	**HR (95% CI)**	**Step**	**χ** ^2^ **change from previous step**
Age at last in‐trial follow‐up, 20 categories	19	Various	0		Various	0		Various	0	
Allocated simvastatin (placebo as reference)	1	1.03 (0.89–1.18)	0		1.04 (0.90–1.20)	0		1.04 (0.91–1.20)	0	
Allocated vitamin (placebo as reference)	1	0.99 (0.86–1.14)	0		1.00 (0.87–1.15)	0		1.00 (0.87–1.15)	0	
Height at screening, per 10 cm	1	0.96 (0.86–1.07)	0		1.04 (0.93–1.16)	0		1.03 (0.92–1.15)	0	
Men	1	1.08 (0.87–1.34)	0		1.00 (0.81–1.23)	0		1.00 (0.81–1.23)	0	
Townsend Index, per unit	1	1.06 (1.03–1.08)	0		1.02 (1.00–1.05)	0		1.02 (1.00–1.04)	0	
Cognitive Z‐score available	1				0.22 (0.16–0.30)	0		0.21 (0.16–0.29)	0	
*APOE ε2/ε3/ε4* genotype available	1							0.75 (0.63–0.89)	0	
Cognitive Z‐score, per ‐0.2 SD	1				1.13 (1.11–1.15)	1	255.1	1.13 (1.11–1.15)	1	254.8
*APOE ε4*, per allele effect	1							1.88 (1.65–2.15)	2	86.1
Follow‐up minus baseline weight sex‐specific fifths										
1	4	1.45 (1.26–1.67)	1	38.3	1.43 (1.25–1.65)	2	36.2	1.38 (1.20–1.59)	3	31.3
2		1.06 (0.92–1.23)			1.06 (0.91–1.23)			1.04 (0.90–1.21)		
3		1.00 (0.87–1.15)			1.00 (0.87–1.15)			1.00 (0.87–1.15)		
4		0.75 (0.62–0.91)			0.72 (0.59–0.87)			0.72 (0.60–0.88)		
5		0.88 (0.73–1.07)			0.87 (0.71–1.05)			0.87 (0.72–1.05)		
Non‐fatal in‐trial transient ischemic attack	1	1.88 (1.37–2.57)	2	13.3	1.79 (1.30–2.45)	3	10.9	1.74 (1.27–2.38)	4	9.9
Insulin at baseline	1				1.55 (1.19–2.01)	4	9.4	1.50 (1.16–1.96)	5	8.3
Diabetes at baseline	1	1.35 (1.16–1.59)	3	11.6						
Low density lipoprotein cholesterol at the end of active run‐in (on statin), per mmol/L	1	1.18 (1.05–1.32)	4	7.8						

*Note*: The factors listed as step 0 were forced into the model. Variables selected at *P*‐value < .005 shown. See Supplementary Information Table S4 for the complete list of factors considered for inclusion in the stepwise models.

Abbreviations: *APOE*, apolipoprotein E; CI, confidence interval; DF, degrees of freedom; HR, hazard ratio; χ^2^ , likelihood ratio test chi‐squared.

### 3.5 Identifying a high dementia risk subset

Table 3 and Supplementary Information Table S6 show the absolute risks of recorded dementia in the hospital records during the first 10 years of post‐trial follow‐up within age, previous disease, and risk groups. Of the 12,975 participants surviving to final in‐trial follow‐up without dementia recorded, 4961 (38%) were aged <67 years and had a low risk of dementia. Among the remaining 8014 participants, 638 (8.0%) had dementia recorded within the 10 years. Among 1712 with weight loss ≥4 kg, dementia was recorded in 9.9% overall, and in 12.8% of the 988 participants who additionally had a cognitive function z‐score < 0 or not available. Rates were higher in *APOE* ε4 carriers, but this identified only a much small proportion of the population (Supplementary Information Table S6). Death before recorded dementia was a competing risk and among those aged ≥67 years occurred in 44.9% overall and in 58.8% of those with weight loss ≥4 kg and cognitive function z‐score < 0 or missing (Supplementary Information Table S6).

**TABLE 3:** Incidence of first recorded dementia in the Heart Protection Study (HPS) during 0‐9 years post‐trial by age at final in‐trial follow‐up and risk group

	**Previous disease (at baseline or in‐trial)**					
	**Cerebrovascular disease**		**Other** ^a^		**Overall**	
**Age at final in‐trial follow‐up, years**	**Dementia/at risk**	**Rate/100 py**	**Dementia/at risk**	**Rate/100 py**	**Dementia/at risk (%)**	**Rate/100 py**
All participants aged ≥67						
Age: 67–71	23/482 (4.8%)	0.59	94/2180 (4.3%)	0.51	117/2662 (4.4%)	0.53
Age: 72–76	59/642 (9.2%)	1.29	185/2388 (7.7%)	1.02	244/3030 (8.1%)	1.07
Age: ≥77	60/498 (12.0%)	1.96	217/1824 (11.9%)	1.78	277/2322 (11.9%)	1.82
Total aged ≥67	142/1622 (8.8%)	1.23	496/6392 (7.8%)	1.02	638/8014 (8.0%)	1.06
Weight loss ≥4 kg						
Age: 67–71	4/103 (3.9%)	0.52	24/376 (6.4%)	0.84	28/479 (5.8%)	0.77
Age: 72–76	16/129 (12.4%)	2.02	46/489 (9.4%)	1.39	62/618 (10.0%)	1.51
Age: ≥77	18/145 (12.4%)	2.52	62/470 (13.2%)	2.33	80/615 (13.0%)	2.37
Total aged ≥67	38/377 (10.1%)	1.67	132/1335 (9.9%)	1.49	170/1712 (9.9%)	1.53
Cognitive z‐score < 0 or missing						
Age: 67–71	17/240 (7.1%)	0.90	61/955 (6.4%)	0.78	78/1195 (6.5%)	0.80
Age: 72–76	49/397 (12.3%)	1.83	133/1205 (11.0%)	1.52	182/1602 (11.4%)	1.59
Age: ≥77	50/334 (15.0%)	2.57	165/1124 (14.7%)	2.30	215/1458 (14.7%)	2.36
Total aged ≥67	116/971 (11.9%)	1.78	359/3284 (10.9%)	1.51	475/4255 (11.2%)	1.57
Weight loss ≥4 kg and cognitive z‐score < 0 or missing						
Age: 67–71	2/50 (4.0%)	0.56	18/163 (11.0%)	1.46	20/213 (9.4%)	1.26
Age: 72–76	11/79 (13.9%)	2.51	31/267 (11.6%)	1.82	42/346 (12.1%)	1.96
Age: ≥77	16/109 (14.7%)	3.15	48/320 (15.0%)	2.78	64/429 (14.9%)	2.87
Total aged ≥67	29/238 (12.2%)	2.23	97/750 (12.9%)	2.08	126/988 (12.8%)	2.11

Abbreviation: py, person years at risk.

^a^ Coronary disease, other occlusive arterial disease, diabetes or hypertension at baseline, excluding those with cerebrovascular disease at baseline or in‐trial.

## 4 DISCUSSION

In a cardiovascular trial population, where most participants had pre‐existing atherosclerotic disease, greater weight loss over 5 years and lower baseline or follow‐up weight, were all predictors of future dementia incidence. A measure of weight change was the strongest predictor of future dementia risk from among the risk factors available in the trial (after allowing for age, sex, randomized trial treatment allocation, height, and Townsend index), in the absence of knowledge of cognitive function or *APOE* genotype. When cognitive function or *APOE* genotype were available, they were the strongest predictors but a measure of weight change was the next strongest predictor.

Weight loss, compared to maintaining a steady weight, was associated with higher dementia risk in a large individual participant meta‐analysis of prospective studies and in recent large studies (>60,000 incident cases of dementia in total).^18^, ^19^, ^35^, ^36^ Weight gain was also associated with higher dementia risk in some studies but less consistently and with some studies suggesting that this association was limited to vascular dementia.^18^ However, data on weight change and vascular dementia incidence were very limited. The *APOE* ε4 genotype is a strong risk factor for dementia and in the present study, *APOE* ε4 genotypes were associated with weight loss. Consistent with this, a recent study in UK Biobank found that genetic risk of dementia began to predict lower BMI from about age 50, further suggesting that weight loss might manifest as an early pathophysiological change associated with dementia.^37^ The mechanisms by which weight loss is associated with increased risk of dementia remain debated: it may be due to reverse causality and pathological changes in the brain,^18^ but alternatively, a role of impaired brain energetics as a causal factor for dementia has gained support.^38^

Dementia trials are often under‐powered.^39^, ^40^ For example, the preDIVA multidomain intervention cluster randomized trial, which included 3500 participants who were in their 70s but not otherwise selected by risk, was powered to detect a 33% proportional reduction in dementia incidence with the intervention over 6 years but observed only 7% incidence of dementia and a non‐significant 8% proportional reduction in risk^41^. It therefore concluded that future studies should select populations at higher risk. Weight loss of at least 4 kg helped identify a group at high risk of dementia in the present study. Among HPS survivors to final in‐trial follow‐up aged ≥67 years, 8.0% had dementia recorded in hospital admissions during the following 10 years of follow‐up. The percentage rose to 9.9% when restricting to those with weight loss of at least 4 kg and to 12.8% when further excluding those with cognitive function z‐score above the overall mean in HPS. In a population at this risk, a randomized trial in 10,000 participants of an intervention over 10 years would have 90% power to detect a 20% proportional reduction in dementia risk at 2*P* < .01 (Supplementary Information Table S1).

Risk scores for dementia have been proposed as a means of identifying higher risk populations for trials. The simple CAIDE risk score ≥6 was used for initial invitation of participants, followed by cognitive testing criteria, in the Finnish Geriatric Intervention Study to Prevent Cognitive Impairment and Disability (FINGER) trial.^42^ A CAIDE score of 6–7 corresponded to a dementia risk of only 2% over 20 years in the original study developing the score and so may only be useful for studies of cognitive function outcomes rather than dementia incidence. The BDSI^33^ and the ANU‐ADRI have been recommended as dementia risk profiling measures in individuals aged 65 and older.^15^ However, none of these three scores or measures included weight loss. Only about half of the terms other than age, sex, and cognitive measures in these scores were available in HPS, and the partial scores from these were not selected as predictive of dementia in HPS in the present analyses.

In a review of scores for dementia risk, about a quarter of the scores included BMI as a risk factor, but none included change in weight or BMI.^16^ As the direction of the association of BMI with dementia varies with length of follow‐up and age,^9^, ^11^, ^17^ single measures of weight or BMI have contributed to risk scores in different ways depending on the context.^33^, ^34^, ^43^, ^44^, ^45^. Scores with more extensive/expensive measures such as genotyping or brain imaging tended to perform slightly better in the reviewed studies.^16^ *APOE* genotype was a strong risk factor in the scores that included it, as found in the present study, and a polygenetic risk score using genome‐wide data in one score^46^ achieved additional predictive power. However, expensive measures may be less feasible for use in large scale contexts. By contrast, weight is easily measured and was available in about two‐thirds of individuals in routine primary care data in a study in the United Kingdom, with a mean interval of 2 years in those with a repeat measure.^47^ Weight loss may reflect a pre‐clinical stage of dementia and therefore may be relevant for the identification of populations for the assessment of any interventions that might slow the progression from pre‐clinical to clinically manifest dementia, but may be particularly relevant for interventions to address brain energy deficits, which are an avenue under investigation.^38^

## 5 LIMITATIONS

The study was limited by only capturing dementia after the scheduled treatment period from hospital admissions and death records. Dementia in hospital admissions are on average 1–2 years later than diagnosis in primary care.^48^ Therefore, the observed rates from hospital admission data at ages ≥67 years may be interpreted as predicted rates at ages ≥65 years. However, routinely collected health data dementia diagnoses have a high positive predictive value and reasonable sensitivity for a clinical diagnosis, which is somewhat better with primary care data.^49^ An analysis in the UK Biobank population where primary care records as well as hospital admission diagnoses were available (Supplementary methods, Supplementary Information Table S7) suggested that, after allowing for a 2 year delay, 20% more cases might be diagnosed in primary care over those from hospital admission diagnoses. Hence, the absolute percentages with dementia diagnoses may be about 2% higher than the observed rates cited above (and in Table 3). Against this, trial participants willing to continue to a further trial would be likely to be somewhat healthier than survivors overall, leading to somewhat lower expected rates. The HPS study largely comprised white British participants; therefore, the generalisability of findings to other high vascular risk populations remains to be evaluated.

## 6 CONCLUSIONS

In a population with pre‐existing occlusive vascular disease or at high vascular risk, after allowing for age, weight loss was the strongest predictor of the incidence of recorded dementia in the absence of a cognitive function measure and *APOE* genotype. Weight loss ≥4 kg and cognitive function below the mean identified a set of participants with a projected >12% risk of dementia diagnosis over 10 years. Such a population risk would provide power to detect a 20% proportional reduction in the incidence of dementia with an intervention in a trial of 10,000 participants over 10 years. Weight loss is a frequently available factor that should be considered in dementia risk prediction scores.

## CONFLICTS OF INTEREST

All authors worked in the Clinical Trial Service Unit & Epidemiological Studies Unit of the Nuffield Department of Population Health at the University of Oxford. The Clinical Trial Service Unit & Epidemiological Studies Unit has a staff policy of not taking any personal payments directly or indirectly from industry (with reimbursement sought only for the costs of travel and accommodation to attend scientific meetings). Support for the present manuscript to all authors came from grants to the University of Oxford from Merck, Roche, the UK Medical Research Council, the British Heart Foundation and Cancer Research UK (see acknowledgements section for full details of funding). P.V., I.H., G.B., M.G., A.O., and M.N. have nothing further to disclose. W.W. reports support from the Chief Scientist's Office and the Alzheimer's Society, membership of a Data Safety Monitoring Board outside the area of this publication and a leadership or fiduciary role on the Stroke editorial board. R.B. reports he is chair of a NIHR funded trial (CHAPS) looking at compression hosiery to prevent post‐thrombotic syndrome (unpaid). R.C. is deputy chair of a not‐for‐profit Clinical Trial Company, Chief Executive of UK Biobank and chair of the Data Monitoring Committee of PROMINENT Data Safety Monitoring Board of Pemafibrate. J.A. reports a grant from Novartis and membership of Data Safety Monitoring Board (unpaid). M.M. reports grants from Novartis and Novo Nordisk. S.P. is statistician to the Data Monitoring Committee of the ORION‐4 trial of inclisiran. S.P. and R.C. are co‐inventors of a genetic test for statin‐related myopathy risk but receive no income from it. Author disclosures are available in the supporting information.

## AUTHOR CONTRIBUTIONS

Richard Bulbulia, Rory Collins, Jane Armitage, and Sarah Parish contributed to data collection and study design. Michelle Nunn and William Whiteley contributed to data collection. Pieter van der Veere, Georgina Buck, Melanie Greenland, Imen Hammami, Alison Offer, and Sarah Parish contributed to statistical analysis. Imen Hammami, Marion Mafham, and Sarah Parish supervised the project. Pieter van der Veere and Sarah Parish drafted the manuscript and all authors contributed to its interpretation and re‐drafting. Georgina Buck and Sarah Parish had full access to all the data in the study and take responsibility for the integrity of the data and the accuracy of the data analysis.

## Supporting information

SUPPORTING INFORMATION

SUPPORTING INFORMATION

## Data Availability

Proposals for data access will be considered by the HPS custodians in accordance with the trial protocol. Procedures for accessing the data are available at: https://www.ndph.ox.ac.uk/files/about/ndph‐data‐access‐policy‐1.pdf.
