# Supplementary material for: Weight loss in a cardiovascular trial population identifies people at future risk of dementia
Source: Alzheimers Dement (Amst). 2022 Aug 30;14(1):e12352. doi: 10.1002/dad2.12352 (PMC9428278; doi:10.1002/dad2.12352)
Supplement: Supplementary file 1 — SUPPORTING INFORMATION [file DAD2-14-e12352-s002.pdf]

Supplementary materials

Table of contents

Supplementary methods		2
Supplementary references		3
Supplementary tables		
Table S1	Power of a randomized trial in 10,000 participants to detect a 20% proportional reduction in dementia incidence with treatment, according to the proportion of control participants getting dementia	4
Table S2	International Classification of Disease (ICD-10) codes used for dementia	5
Table S3	Boundaries of the fifths of weight and body mass index measures within each sex	6
Table S4	Factors available for selection in the stepwise model of incidence of first recorded dementia post-trial	7
Table S5	Availability of factors other than age, sex and cognitive function terms in three published dementia risk scores in Heart Protection Study (HPS) data	9
Table S6	Incidence of first recorded dementia and death before recorded dementia in the Heart Protection Study (HPS) during 0-9 years post-trial by age at final in-trial follow-up and risk group	11
Table S7	Dementia recording in secondary and primary care data among UK biobank participants with linkage to both sources	12
Supplementary figures		
Figure S1	Distribution of weight change between baseline and follow-up	13
Figure S2	Associations of different body mass index (BMI) measures with the incidence of first recorded dementia post-trial, after standard adjustment	14
Figure S3	Associations of different systolic blood pressure (SBP) measures with the incidence of first recorded dementia post-trial, after standard adjustment	15
Figure S4	Association of weight change with the incidence of first recorded dementia post-trial, within subgroups by age at final follow-up, follow-up period and *APOE* genotype	16
Figure S5	Association of *APOE* genotype with the incidence of first recorded dementia post-trial, cognitive z-score and weight change	17

Supplementary methods

To estimate the number of dementia cases not identified through linkage to hospital admissions (secondary care) that might be identified through primary care data, a support analysis was conducted using data from 230,685 participants in the UK Biobank prospective study who were linked to both NHS Digital Hospital Episode Statistics (as available in the Heart Protection Study) and Primary care data [1, 2]. Follow-up data were censored at 14 June 2016, the last date that all sources of primary and secondary care data appeared to be complete (in data available for non-COVID-19 research), giving a mean follow-up of 7.3 years. First occurrence of dementia in primary care was defined using events in the UK Biobank first occurrence dataset where that earliest occurrence was from primary data records. This dataset defined dementia as ICD-10 codes F00, F01, F02, F03 or G30 (which is very similar to the definition used in the present study). Dementia in secondary care/death records was defined using the same codes. Participants with dementia first recorded prior to baseline were excluded from the analysis (n=123). The subset of participants with myocardial infarction, stroke, transient ischaemic attack, angina, medically lowered blood pressure, revascularisation or diabetes at baseline were regarded as 'cardiovascular trial like' and therefore should be broadly similar to those in the Heart Protection Study.

The frequency of dementia in secondary care data, in primary care data as well, and alone, and in primary care data alone with at least 2 years of subsequent follow-up, was tabulated by age (<60, ≥60 years) and cardiovascular status.

Supplementary references

[1] UK Biobank [accessed 15 February 2022]. Available from: http://www.ukbiobank.ac.uk/.

[2] UK Biobank Primary Care Linked Data [accessed 15 February 2022]. Available from: https://biobank.ctsu.ox.ac.uk/crystal/crystal/docs/primary_care_data.pdf.

[3] P. Armitage, G. Berry, J.N.S. Matthews, Statistical methods in medical research. Oxford: Blackwell Science; 2002.

[4] K.J. Anstey, N. Cherbuin, P.M. Herath, Development of a new method for assessing global risk of Alzheimer's disease for use in population health approaches to prevention. Prev Sci 2013;14:411-21.

[5] D.E. Barnes, A.S. Beiser, A. Lee, K.M. Langa, A. Koyama, S.R. Preis, et al., Development and validation of a brief dementia screening indicator for primary care. Alzheimers Dement 2014;10:656-65 e1.

[6] M. Kivipelto, T. Ngandu, T. Laatikainen, B. Winblad, H. Soininen, J. Tuomilehto, Risk score for the prediction of dementia risk in 20 years among middle aged people: a longitudinal, population-based study. Lancet Neurol 2006;5:735-41.

Table S1: Power of a randomized trial in 10,000 participants to detect a 20% proportional reduction in dementia incidence with treatment, according to the proportion of control participants getting dementia.

	Numbers of dementia cases and power assuming a 20% proportional reduction with active treatment		
**Assumed % of controls getting dementia**	**Control N dementia/ N total**	**Active N dementia/ N total**	**Power to detect effect at 2P<0.01**
10%	500/5000	400/5000	82%
12%	600/5000	480/5000	90%
15%	750/5000	600/5000	96%

Calculations based on a comparison of the proportions [3] implemented using R.

Table S2: International Classification of Disease (ICD-10) codes used for dementia

ICD-10	Dementia
	Alzheimer’s disease
F00	Dementia in Alzheimer's disease
F000A	Dementia in Alzheimer's disease with early onset
F001A	Dementia in Alzheimer's disease with late onset
F002A	Dementia in Alzheimer's disease, atypical or mixed type
F009A	Dementia in Alzheimer's disease, unspecified
G30	Alzheimer’s disease
G30.0	Alzheimer’s disease with early onset
G30.1	Alzheimer’s disease with late onset
G30.8	Other Alzheimer disease, unspecified
G30.9	Alzheimer's disease unspecified
	Vascular dementia
F01	Vascular dementia
F010	Vascular dementia of acute onset
F011	Multi-infarct dementia
F012	Subcortical vascular dementia
F013	Mixed cortical and subcortical vascular dementia
F018	Other vascular dementia
F019	Vascular dementia, unspecified
I67.3	Binswanger's disease
	Unspecified dementia type
F03X	Unspecified dementia
F051	Delirium superimposed on dementia

Table S3: Boundaries of the fifths of weight and body mass index measures within each sex

Weight/BMI measure	Sex-specific fifth	Weight measure range (kg)		BMI measure range (kg/m^2)	
		Men	Women	Men	Women
Follow-up minus baseline					
	1	-33 to -4	-34 to -4	-11.15 to -1.05	-12.80 to -1.54
	2	-3 to -1	-3 to -1	-1.04 to -0.27	-1.53 to -0.31
	3	0 to 2	0 to 2	0.00 to 0.68	0.00 to 0.81
	4	3 to 5	3 to 5	0.68 to 1.72	0.81 to 2.11
	5	6 to 40	6 to 26	1.73 to 14.34	2.11 to 11.57
Percentage change from baseline to follow-up (%)					
	1	-32.91 to -3.96	-31.78 to -5.41	-32.91 to -3.96	-31.78 to -5.41
	2	-3.95 to -0.76	-5.38 to -0.77	-3.95 to -0.76	-5.38 to -0.77
	3	0.00 to 2.61	0.00 to 3.03	0.00 to 2.61	0.00 to 3.03
	4	2.63 to 6.35	3.08 to 7.84	2.63 to 6.35	3.08 to 7.84
	5	6.36 to 49.38	7.89 to 50.00	6.36 to 49.38	7.89 to 50.00
Baseline					
	1	44 to 71	37 to 60	15.67 to 24.22	14.33 to 23.92
	2	72 to 77	61 to 67	24.24 to 26.08	23.92 to 26.31
	3	78 to 83	68 to 74	26.09 to 27.77	26.35 to 28.91
	4	84 to 91	75 to 83	27.77 to 30.04	28.93 to 32.47
	5	92 to 191	84 to 141	30.04 to 64.56	32.47 to 61.51
Follow-up					
	1	40 to 71	32 to 60	15.85 to 24.25	13.32 to 24.00
	2	72 to 78	61 to 68	24.26 to 26.23	24.00 to 26.62
	3	79 to 84	69 to 74	26.25 to 28.09	26.63 to 29.39
	4	85 to 93	75 to 85	28.09 to 30.64	29.39 to 33.26
	5	94 to 170	86 to 138	30.67 to 57.46	33.27 to 56.30

Abbreviations: BMI = body mass index

Table S4: Factors available for selection in the stepwise model of incidence of first recorded dementia post-trial

Factor
Age at last in-trial follow-up (20 groups)
Sex
Townsend Index*
Allocated simvastatin
Allocated vitamins
Height at screening
Smoking at baseline
Current smoker
Smoking status (current, ex-, never smoker)
Alcohol at baseline
Drinks alcohol now
Alcohol units/week
Grouped alcohol units/week (0,<20,20+)
Medication at baseline
Angiotension converting enzyme inhibitors/ angiotensin receptor blocker
Aldosterone antagonist
Alpha blocker
Aspirin
Beta blocker
Bronchial dilator
Calcium channel blocker
Diuretic
Insulin
Nitrate
Non-steroidal anti-inflammatory drugs / COX-2 inhibitors
Warfarin
Other anti-platelet medication
Oral hypoglycaemics
Blood measurements at screening (off statin) †
Albumin
Creatinine
log(Creatinine)
Estimated glomular filtration rate
C-reactive protein
log(C-reactive protein)
Blood measurements at the end of active run-in (on statin) †
Apolipoprotein A1
Apolipoprotein B
Low Density Lipoprotein (LDL) cholesterol
High Density Lipoprotein (HDL) cholesterol

Factor
Blood measurements at the end of active run-in (on statin) †, continued
Total cholesterol
Triglycerides
Baseline prior disease
Cerebrovascular disease
Peripheral vascular disease
Prior coronary disease (myocardial infarction; other coronary heart disease; none)
Hospitalisation for angina
Treated hypertension
Diabetes
Non-fatal in-trial events
Stroke
Transient ischaemic attack
Myocardial infarction
Heart failure
New onset diabetes
Cancer
APOE ε2/ε3/ε4 genotype
Available
ε2 allele count
ε4 allele count
Final in-trial follow-up measurements
Cognitive Z-score available
Cognitive Z-score
Follow-up minus baseline sex-specific fifths
Weight
Systolic blood pressure ‡
Diastolic blood pressure ‡
Published risk scores§
ANU-ADRI
BDSI
CAIDE

* Imputed as the median value (-1.28) in the 39 cases with missing Townsend Index

† Imputed as the sex-specific median value in cases with missing values: C-reactive protein / log(C-reactive protein) (n=1,324), apolipoprotein A1 (n=7), apolipoprotein B (n=7), LDL cholesterol (n=8), HDL cholesterol (n=8), total cholesterol (n=7), triglycerides (n=7)

‡ Imputed as the middle fifth in cases with missing baseline or final in-trial systolic (n=10) and diastolic (n=11) blood pressure

§ Derived from the available terms of the risk scores other than age, sex and cognitive function. ANU-ADRI = Australian National University Alzheimer's Disease Risk Index [4], BDSI = Brief Dementia Screening Indicator [5], CAIDE = Cardiovascular Risk Factors, Aging, and Incidence of Dementia Risk Score [6]

Table S5: Availability of factors other than age, sex and cognitive function terms in three published dementia risk scores in Heart Protection Study (HPS) data

	ANU-ADRI	BDSI	CAIDE	Availability in HPS at final in-trial follow-up (or substitute time point)
Body mass index (kg/m^2)	Age <60 years:<br>18.5-24.99 (0 points)<br>25-29.99 (2 points)<br>≥30 (5 points)<br>Age ≥60 years (0 points)	<18.5 (8 points)	≤30 (0 points)<br>>30 (2 points)	Available
Diabetes	Self report, use of anti-diabetic medication or laboratory test (3 points)	Type 2 (3 points)		Available - type 1 and type 2 not distinguished, but most likely type 2
Ever had a stroke		Yes (6 points)		Available
Total cholesterol (mmol/L)	Age <60 years:<br><6.2 (0 points)<br>>6.2 (3 points)<br>Age ≥60 years (0 points)		≤6.5 (0 points)<br>>6.5 (2 points)	Available - on-statin total cholesterol at randomization used
Smoking	Never (0 points)<br>Ever (1 point)<br>Current (4 points)			Available at trial entry
Alcohol intake	No alcohol (0 points)<br>Light-moderate (-3 points)<br>Heavy (0 points)			Available at trial entry
Systolic blood pressure (mmHg)			≤140 (0 points)<br>>140 (2 points)	Available
Education (years)	>11 (0 points)<br>8-11 (3 points)<br><8 (6 points)	<12 (9 points)	≥10 (0 points)<br>7-9 (2 points)<br>0-6 (3 points)	Not available
Depressive symptoms	No (0 points)<br>Yes (2 points)	No (0 points)<br>Yes (6 points)		Not available

	ANU-ADRI	BDSI	CAIDE	Availability in HPS at final in-trial follow-up (or substitute time point)
Traumatic brain injury	No (0 points)<br>Yes (4 points)			Not available
Physical activity	Lowest activity (0 points)<br>Medium activity (-2 points)<br>Highest activity (-3 points)		Active (0 points)<br>Inactive (1 point)	Not available
Social engagement	Highest (0 points)<br>Medium to high (1 point)<br>Low to medium (4 points)<br>Lowest (6 points)			Not available
Fish intake (portions per week)	<0.25 (0 points)<br>0.25-2 (-3 points)<br>2-4 (-4 points)<br>>4 (-5 points)			Not available
Pesticide exposure	Never (0 points)<br>Ever (2 points)			Not available

Abbreviations: ANU-ADRI = Australian National University Alzheimer's Disease Risk Index [4], BDSI = Brief Dementia Screening Indicator [5], CAIDE = Cardiovascular Risk Factors, Aging, and Incidence of Dementia Risk Score [6], M=Male, F=Female

Table S6: Incidence of first recorded dementia and death before recorded dementia in the Heart Protection Study (HPS) during 0-9 years post-trial by age at final in-trial follow-up and risk group

	Dementia		Death before dementia	
**Age at final in-trial follow-up, years**	**At risk (%)**	**Rate /100 py**	**At risk (%)**	**Rate /100 py**
All participants				
Age: <67	64/4961 (1.3%)	0.14	956/4961 (19.3%)	2.13
Age: 67-71	117/2662 (4.4%)	0.53	889/2662 (33.4%)	4.01
Age: 72-76	244/3030 (8.1%)	1.07	1364/3030 (45.0%)	6.01
Age: ≥77	277/2322 (11.9%)	1.82	1348/2322 (58.1%)	8.84
Total aged ≥67	638/8014 (8.0%)	1.06	3601/8014 (44.9%)	5.99
Weight loss ≥4kg				
Age: 67-71	28/479 (5.8%)	0.77	210/479 (43.8%)	5.77
Age: 72-76	62/618 (10.0%)	1.51	343/618 (55.5%)	8.36
Age: ≥77	80/615 (13.0%)	2.37	413/615 (67.2%)	12.25
Total aged ≥67	170/1712 (9.9%)	1.53	966/1712 (56.4%)	8.69
Cognitive z-score <0 or missing				
Age: 67-71	78/1195 (6.5%)	0.80	403/1195 (33.7%)	4.13
Age: 72-76	182/1602 (11.4%)	1.59	749/1602 (46.8%)	6.55
Age: ≥77	215/1458 (14.7%)	2.36	865/1458 (59.3%)	9.48
Total aged ≥67	475/4255 (11.2%)	1.57	2017/4255 (47.4%)	6.65
Weight loss ≥4kg and cognitive z-score <0 or missing				
Age: 67-71	20/213 (9.4%)	1.26	90/213 (42.3%)	5.67
Age: 72-76	42/346 (12.1%)	1.96	200/346 (57.8%)	9.35
Age: ≥77	64/429 (14.9%)	2.87	291/429 (67.8%)	13.03
Total aged ≥67	126/988 (12.8%)	2.11	581/988 (58.8%)	9.75
Weight loss ≥4kg and cognitive z-score <0 or missing and *APOE ε4* carrier*				
Age: 67-71	8/54 (14.8%)	2.03	21/54 (38.9%)	5.33
Age: 72-76	17/84 (20.2%)	3.30	41/84 (48.8%)	7.96
Age: ≥77	25/120 (20.8%)	3.79	74/120 (61.7%)	11.21
Total aged ≥67	50/258 (19.4%)	3.19	136/258 (52.7%)	8.67

Abbreviations: py = person years at risk

The data line for age<67 years is shown in grey because these participants have not been included in the totals below (as their rate of dementia is too low).

* Among the 71.5% of the 8014 participants in this table that had *APOE* genotyping

Table S7: Dementia recording in secondary and primary care data among UK biobank participants with linkage to both sources

Subgroup	Number of participants	Mean (SE) years follow-up*	Number of participants with dementia: Secondary care/death records: All	First in primary care >2 years earlier	First in primary care ≤2 years earlier	No preceding record in primary care‡	Primary care only	In primary care with at least 2 years subsequent secondary care/death follow up and not in secondary care/death records within 2 years as a proportion of all secondary care/death record dementias
Cardiovascular trial like†								
Age<60 years	22466	7.3 (0.007)	44	1	7	36	11	5/44 (11%)
Age≥60 years	38536	7.2 (0.006)	362	20	63	279	118	67/362 (19%)
All								
Age<60 years	131164	7.4 (0.003)	105	4	15	86	32	20/105 (19%)
Age≥60 years	99398	7.2 (0.003)	664	40	88	536	228	136/664 (20%)
Total	230562	7.3 (0.002)	769	44	103	622	260	156/769 (20%)

* Follow-up is censored at the earliest of loss to follow-up from secondary care, primary care or death.

† With prior myocardial infarction, stroke, transient ischaemic attack, angina, medically lowered blood pressure, revascularisation or diabetes at baseline.

‡In this table, primary care data are taken from the first occurrence data fields whereas secondary care/death record data are taken from the hospital inpatient data and/or death certificates. Of 1029 dementias recorded in the first occurrence data, 267 were from primary care only, 140 were from primary care and other sources, 539 were from hospital admissions data only, 73 were from hospital admissions and other sources, 9 were from death records only, 1 was from death records and other sources.

Age is at baseline. SE=Standard error

Overall 769 cases of dementia were identified in secondary care and a further 260 were identified only in primary care. 156 cases identified in primary care with at least 2 years subsequent secondary care/death follow-up had not been identified in secondary care/death records within 2 years. Thus, an additional 156/769 (20%) of dementia cases may remain undetected by hospital admission diagnoses 2 years after primary care diagnosis. The pattern was similar in the different age and cardiovascular status strata.

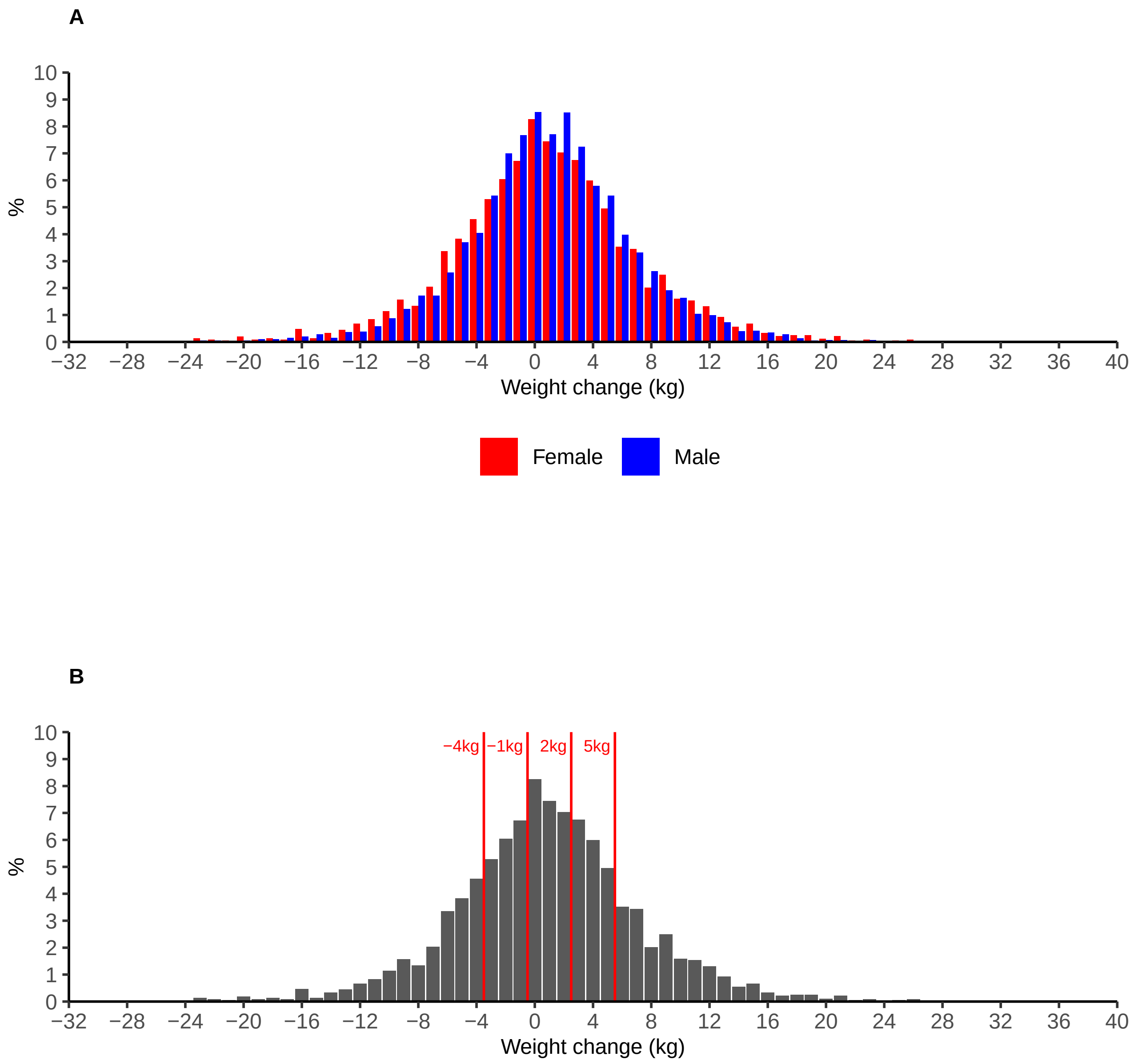

Figure S1: Distribution of weight change between baseline and follow−up. (A) By sex (B) Overall, including boundaries for fifths of weight change.

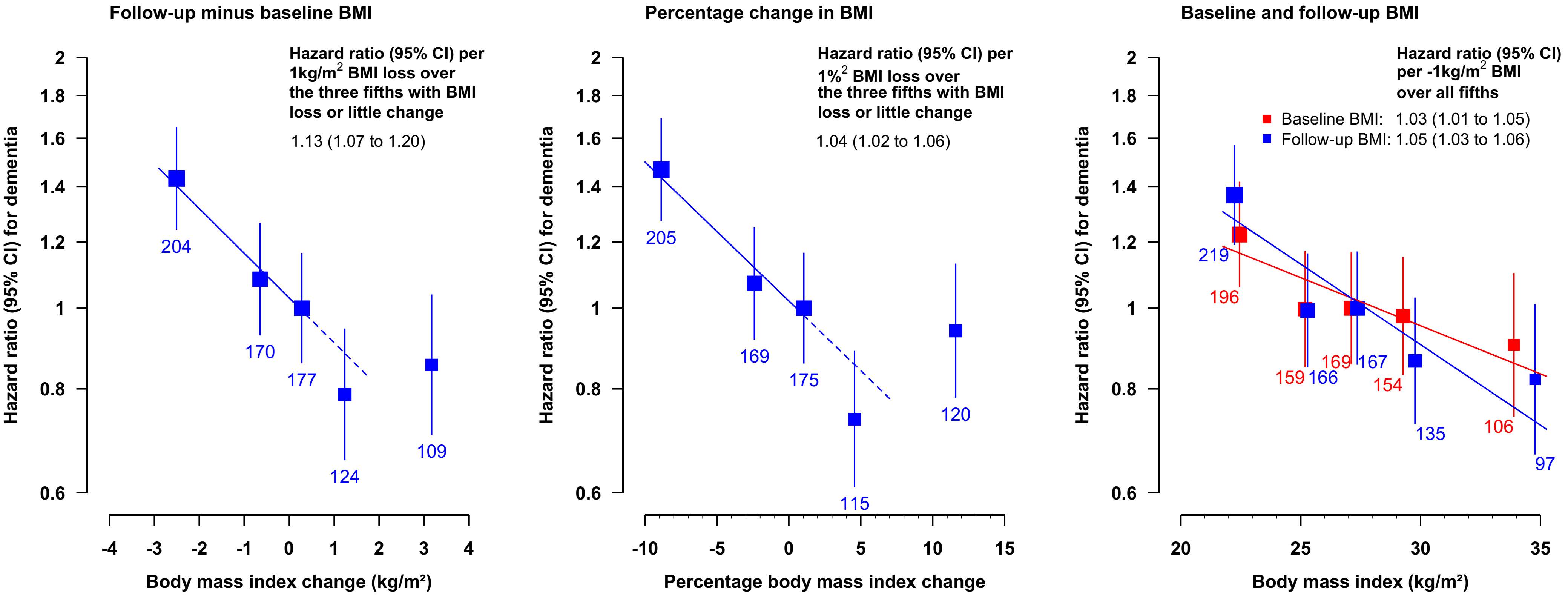

Comparison	Absolute BMI change		Percentage BMI change		Baseline BMI		Follow-up BMI	
	χ^2	***P* value**	χ^2	***P* value**	χ^2	***P* value**	χ^2	***P* value**
Heterogeneity across fifths of BMI measure (4 df)	33.9	<0.0001	38.6	<0.0001	8.1	0.09	25.5	<0.0001
Trend across fifths of BMI measure (1 df)*	28.4	<0.0001	25.2	<0.0001	6.1	0.01	20.5	<0.0001
Non-linearity across fifths of BMI measure (3 df)	5.5	0.14	13.4	0.004	2.0	0.57	4.9	0.18
Trend over lowest 3 fifths of BMI measure (1 df)*	13.7	0.0002	15.0	0.0001	4.3	0.04	10.6	0.001
Lowest versus higher fifths of BMI measure (1 df)	25.0	<0.0001	27.7	<0.0001	7.3	0.007	21.7	<0.0001

Table heading: χ^2 statistics and p-values for the strengths of association of BMI measures with dementia risk

Figure S2: Associations of different body mass index (BMI) measures with the incidence of first recorded dementia post-trial, after standard adjustment. Number of dementia cases shown below each square. The lines displayed in the panels reflect data-driven summaries of the observed patterns. In the first two panels, the solid line shows the trend over the three fifths with BMI loss or little change and the dotted line shows the continuation of this line. *Using the mean BMI measure in each group as exposure dose

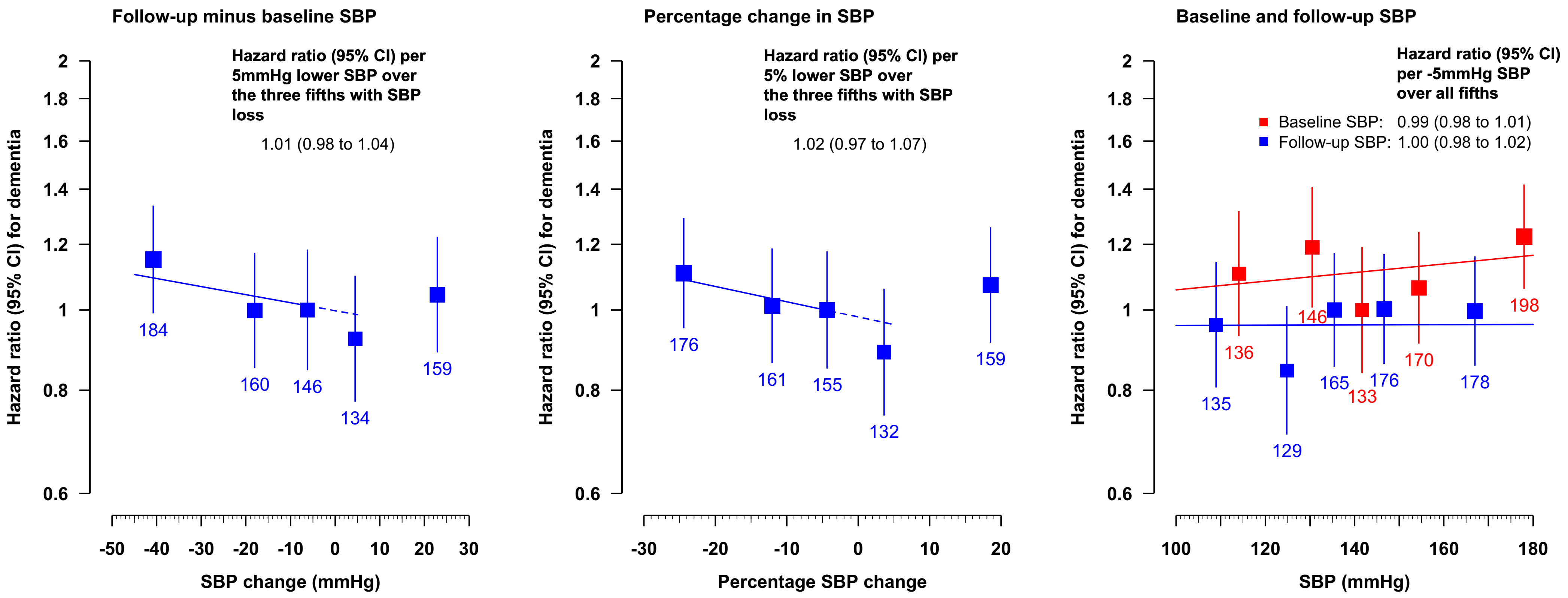

Comparison	Absolute SBP change χ^2	Absolute SBP change ***P* value**	Percentage SBP change χ^2	Percentage SBP change ***P* value**	Baseline SBP χ^2	Baseline SBP ***P* value**	Follow-up SBP χ^2	Follow-up SBP ***P* value**
Heterogeneity across fifths of SBP measure (4 df)	4.1	0.39	4.2	0.38	4.3	0.36	3.0	0.56
Trend across fifths of SBP measure (1 df)*	1.4	0.24	0.4	0.54	0.6	0.45	0.7	0.39
Non-linearity across fifths of SBP measure (3 df)	2.7	0.44	3.8	0.28	3.8	0.29	2.3	0.52
Trend over lowest 3 fifths of SBP measure (1 df)*	2.0	0.16	0.9	0.33	0.4	0.52	0.1	0.71
Lowest versus higher fifths of SBP measure (1 df)	3.0	0.08	1.6	0.21	0.0	0.89	0.0	0.95

Table heading: **χ^2 statistics and p-values for the strengths of association of SBP measures with dementia risk**

Figure S3: Associations of different systolic blood pressure (SBP) measures with the incidence of first recorded dementia post-trial, after standard adjustment. The ten participants (0.08%) with missing baseline or follow-up SBP are excluded from the analysis. Number of dementia cases shown below each square. The lines displayed in the panels reflect data-driven summaries of the observed patterns. In the first two panels, the solid line shows the trend over the three fifths with SBP loss and the dotted line shows the continuation of this line. *Using the mean SBP measure in each group as exposure dose

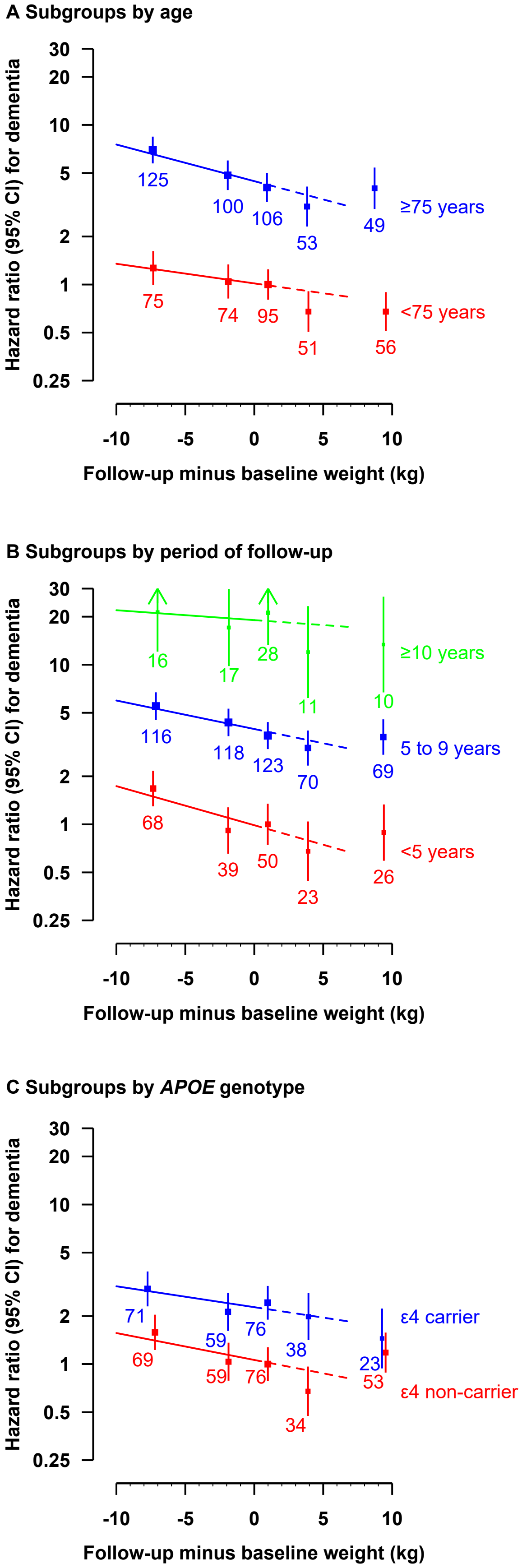

	N participants in the lowest three fifths of weight change	Hazard ratio for dementia per 1kg weight loss across the lowest three fifths of weight change (95% CI) and p-value for heterogeneity	Hazard ratio for dementia in the lowest versus higher fifths of weight change (95% CI) and p-value for heterogeneity
**Age at final follow-up**			
≥75 years	2517	1.05 (1.03 to 1.08)	1.72 (1.40 to 2.12)
<75 years	5635	1.03 (1.00 to 1.06)	1.49 (1.15 to 1.92)
		p=0.21	p=0.38
**Years from final follow-up**			
≥10 years	4608	1.01 (0.95 to 1.09)	1.29 (0.74 to 2.22)
5 to 9 years	6468	1.04 (1.02 to 1.07)	1.51 (1.22 to 1.86)
<5 years	8152	1.06 (1.02 to 1.09)	1.89 (1.41 to 2.53)
		p=0.52	p=0.33
***APOE* genotype**			
ε4 carrier	1900	1.03 (1.00 to 1.06)	1.42 (1.08 to 1.86)
ε4 non-carrier	3822	1.04 (1.01 to 1.07)	1.62 (1.24 to 2.13)
		p=0.72	p=0.49

Figure S4: Association of weight change with the incidence of first recorded dementia post-trial, within subgroups by age at final follow-up, follow-up period and *APOE* genotype. Number of dementia cases shown below each square in the left hand panels. The right hand panels give the values and confidence intervals of the slopes plotted in the corresponding left hand panels and also the hazard ratios for dementia in the lowest versus higher fifths of weight change and their confidence intervals.

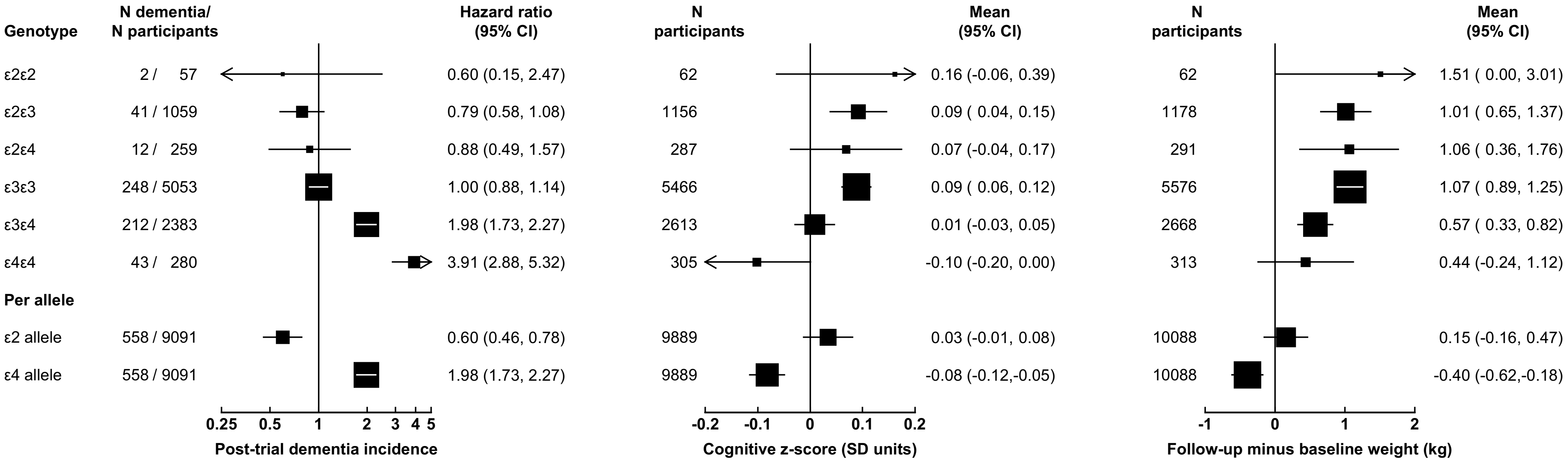

Figure S5: Association of *APOE* genotype with the incidence of first recorded dementia post-trial, cognitive z-score and weight change.
